# Peculiar Antipathies

**Published:** 1884-07

**Authors:** 


### PECULIAR ANTIPATHIES.

The celebrated Erasmus, though a native of Rotterdam, had such an aversion to fish that the smell of it threw him into a fever. Ambroise Paré had a patient who could never see an eel without fainting, and another who would fall into convulsions at the sight of a carp. What would have been the effect of an electric eel on these gentlemen? Joseph Scaliger and others could never drink milk. Gardan was disgusted at the sight of eggs. A King of Poland and a Secretary of France bled at the nose when they looked at apples. Henry III, of France, and many others had a great aversion to cats, mice, spiders, etc. A great huntsman in Hanover, who would attack a wild boar valiantly, always fainted at the sight of roasted pig if he had not time to run away. Amatus Lusitanus knew a person who fainted whenever he saw a rose, and always kept his house when they were in bloom. Scaliger mentions the same about lilies, and Bayle about honey. Bayle himself turned pale at the sight of water-cresses; Tycho-Brahé fainted at the sight of a fox; Henry III, of France, at that of a cat; Marshal d'Albret at a pig. A lady, wonderful enough, could not endure the feel of silk or satin. A man, not so strangely, was known to faint whenever he heard a servant sweeping. Nicanor swooned whenever he heard a bagpipe; Bayle fainted when he heard the splashing of water.—*Med. Record.*

When one bleeds from the nose on looking at apples, or falls into convulsions at the sight of a carp—of what potency is the agent which so affects him? Possibly it *is* above the twelfth decimal.

